# Clinical Performance of Subperiosteal Implants in the Full-Arch Rehabilitation of Severely Resorbed Edentulous Jaws: A Systematic Review and Metanalysis

**DOI:** 10.3390/dj13060240

**Published:** 2025-05-28

**Authors:** Luis Sánchez-Labrador, Santiago Bazal-Bonelli, Fabián Pérez-González, Tomás Beca-Campoy, Carlos Manuel Cobo-Vázquez, Jorge Cortés-Bretón Brinkmann, José María Martínez-González

**Affiliations:** 1 Department of Dental Clinical Specialties, Faculty of Dentistry, Complutense University of Madrid, Plaza Ramón y Cajal S/N, 28040 Madrid, Spain; luissanc@ucm.es (L.S.-L.); sbazal@ucm.es (S.B.-B.); fabianpe@ucm.es (F.P.-G.); cmcobo@ucm.es (C.M.C.-V.); jmargo@ucm.es (J.M.M.-G.); 2 Independent Researcher, 28006 Madrid, Spain; doctorbeca@gmail.com; 3 Surgical and Implant Therapies in the Oral Cavity Research Group, Complutense University of Madrid, 28040 Madrid, Spain

**Keywords:** dental implant, surgical dental prostheses, dental materials, systematic review

## Abstract

**Background/Objectives**: Subperiosteal implants (SPIs) were first used in the 1940s, but due to their complications and the rise of dental implants, they were discontinued. Thanks to new technologies and new materials, nowadays they are being used again and studied as a treatment for severe bone defects. This review analyzes the clinical results—survival rates and complications—of SPIs used to support full arch rehabilitations of severely resorbed maxillae and mandibles, comparing the outcomes resulting from implant placement conducted in one or two surgical interventions. **Methods**: An automated search was conducted in four databases (Medline/Pubmed, Scopus, Web of Science, and Cochrane Library), as well as a manual search for relevant clinical articles published before 28 February 2025. The review included human studies with at least four patients, in which SPIs were placed to restore full-arch edentulous maxillae and mandibles. Quality of evidence was evaluated using the Newcastle–Ottawa Quality Assessment Scale and the Joanna Briggs Institute Critical Appraisal tool. **Results**: A total of 14 studies met the inclusion criteria and were included for analysis, including 958 patients and 973 SPIs. The survival rate was 100% when one surgical intervention was performed and 85% when two interventions were performed after 4–38 months and 3–22 years follow-up, respectively. **Conclusions**: SPIs would appear to offer a good alternative for patients with severe bone atrophies, especially SPIs fabricated using digital techniques in a single step, presenting promising survival rates and a low complication rate, although more randomized clinical trials with long-term follow-up are needed.

## 1. Introduction

Although prosthetic rehabilitations supported by endosseous dental implants achieve predictable outcomes when treating partially and totally edentulous patients, they demand sufficient quantity and quality of bone [1]. A minimum amount of soft tissue around implants is also required to safeguard peri-implant health [2].

Unless extensive regenerative surgeries are performed, endosseous implants may not be a possibility for those patients who present severe bone atrophies. These regenerative surgeries may take the form of inlay/onlay bone blocks, guided bone regeneration (GBR), a split crest technique, bone distraction and/or sinus lift augmentations. Such techniques suffer higher rates of complication, morbidity, and require longer treatment times and higher levels of professional skill [3,4,5,6,7]. Other options are available that can overcome these problems such as short, tilted, or narrow implants; pterygomaxillary implants; zygomatic implants; or subperiosteal implants (SPIs) [1,2,3,4,5,6,7,8].

Concerning the latter option, while SPIs appear to be in vogue at the present time, they were first developed in Sweden as early as the 1940s. Initially, they were manufactured from chrome-cobalt or titanium alloys or even self-curing resin [1,9,10,11]. But the early SPIs required a complex manufacturing process and were difficult to place as two surgeries were needed (the first to replicate the bone anatomy with a conventional impression taking, the second to place the SPI), making adaptation difficult, which could trigger a range of complications, especially infections resulting from poor adaptation [1,12].

Thanks to advances in biomaterials, such as new Ti alloys or polyether-ether-ketone or PEEK, and imaging techniques (computed tomography, stereolithography, intraoral scanners) we have seen a revival of these older techniques, as the first surgery for taking impressions of the recipient bed can be eliminated, making this a more viable and predictable technique for the rehabilitation of atrophic jaws; therefore, nowadays only a single surgery is necessary to place the SPIs. SPIs are placed between the periosteum and the residual alveolar bone, using screws to provide stability; they are covered by mucosa [1,3]; transmucosal elements project through the mucosa into the oral cavity, connecting the implant and prosthesis [1,12,13,14,15]. Among the benefits of SPIs, the following can be highlighted: the elimination of morbidity in the bone donor site in cases of limited bone availability, the reduction of surgical time [15]. In addition, SPIs also offer a treatment option for patients with extreme bone defects due to treatment of oncological diseases or trauma [10,16]. On the other hand, the digital resources (devices and software) necessary to design and manufacture SPIs in a single surgery are not accessible to all professionals, and their clinical performance is not yet well documented.

Due to the increase, in recent years, in the use of SPIs, thanks to new technologies and materials, we believe it is important to know whether SPIs in which only one surgery is used for their placement have better survival rates and fewer complications than the old SPIs in which it was necessary to perform a first surgery to take a measurement of the bone and a second surgery to place the SPIs. In addition, as far as we are aware, no previous systematic review (SR) with meta-analysis has evaluated the clinical performance of SPIs in the full-arch rehabilitations of severely resorbed edentulous jaws. Therefore, the aims of this review were to evaluate the clinical performance of SPIs in terms of survival rate and associated complications, and to compare outcomes between implants placed in one and two surgical procedures. It is hypothesized that SPIs placed in a single surgery will have a longer survival and fewer complications.

## 2. Materials and Methods

This systematic review followed PRISMA (Preferred Reporting Items for Systematic Review and Meta-Analyses) statement guidelines and was registered in the International Prospective Register of Systematic Reviews (PROSPERO; Reg. no. CRD42022372736).

The sections of the PICO(s) question (Population, Intervention, Comparison, Outcome and study design) were as follows:

- Population (P): Edentulous patients with severe atrophy of the jaws restored with subperiosteal implants.
- Intervention (I): SPIs supporting full-arch rehabilitations placed in a single surgery.
- Comparison (C): SPIs placed in two surgical procedures.
- Outcome (O): Clinical performance in terms of survival and complications.
- Study design (s): Clinical studies with a minimum sample size of four patients.

Therefore, the PICO question remains: “In edentulous patients with severe atrophy of the jaws restored with subperiosteal implants, what is the clinical performance (in terms of survival rate and complications) of SPIs differentiating between single-phase and two-stage procedures?

### 2.1. Eligibility Criteria

#### 2.1.1. Inclusion Criteria

- Randomized controlled clinical trials, cohort studies, case-control studies, cross-sectional studies.
- Case series.
- SPI placement in one or two surgical phases.
- Follow-up continuing until (at least) the time of prosthetic restoration.
- Articles published up to and including 28 February 2025.

#### 2.1.2. Exclusion Criteria

- Case reports.
- Animal studies.
- In vitro studies.
- Insufficient information about SPI placement.

### 2.2. Type of Intervention and Comparison

All the articles selected for review included four or more patients receiving SPIs through one or two surgical procedures.

### 2.3. Information Sources and Search Strategy

Four databases underwent an automatic search: The National Library of Medicine (MEDLINE/Pubmed); Web of Science, the Cochrane Library, and Scopus. The search aimed to identify all studies published in English, Spanish or German up to and including 28 February 2025, applying varying combinations of the following search terms: “dental implant”, “subperiosteal implant”, “edentulous patient”, and “survival rate” (Supplementary Material Table S1). A manual search was also carried out in oral surgery, periodontics, and oral implantology journals for any additional articles, and in the reference sections of the papers identified in the automated database search.

### 2.4. Selection Process and Screening Methods

Two reviewers (L.S.L. and S.B.B) independently screened the titles and abstracts of the articles found in searches. They then read the full manuscripts of all studies meeting the inclusion criteria, as well as others with insufficient information in the title and abstract to take a decision as to their relevance, before making a final selection of the studies to be included for review. If any disagreement arose, it was resolved through discussion with the third reviewer (J.C.B.-B.). RefWorks Reference Management Software 2.0 (Ex Libris, Jerusalem, Israel) was employed to identify duplicate references in the electronic databases. If more than one study investigated the same patient cohort, the article with the longest follow-up period was selected. A percentage of agreement and kappa correlation coefficient were calculated to assess inter-reviewer reliability in the selection process.

### 2.5. Data Collection and Data Items

Primary outcomes were the survival rate of SPIs supporting full-arch rehabilitations and differences in outcome between SPI placement performed in one surgery and two surgeries. Secondary outcomes were any associated complications. The two reviewers performed data extraction in duplicate. When data were incomplete or missing from a text, the reviewers contacted the authors. If this could not be done, data were excluded. The data extracted were authors, year of publication, journal, study design, number of patients, mean patient age, follow-up time, number of implants and their locations, number of interventions, prosthesis type, opposite dentition, and implants/prosthesis survival rates.

### 2.6. Study Risk of Bias Assessment, Reporting Bias Assessment and Certainty Assessment

The Newcastle–Ottawa scale (NOS) was applied to assess risk of bias in cohort studies. The NOS considers three key features: selection of study groups, comparability of participants, and outcome. The maximum score is nine points [17] or eight points for cohort studies with a single exposure. Studies were classified as good, fair, or poor-quality (GQ, FQ or PQ) using the score algorithm recommended by the Agency for Healthcare Research and Quality [18].

The Joanna Briggs Institute Critical Appraisal tool was used to assess risk of bias in case series. This applies a checklist of 10 questions, some relating to risk of bias, and others that aim to ensure the quality of reporting and statistical analysis. Each negative response reduces the score awarded for overall quality [19].

### 2.7. Effect Measures and Synthesis Methods

The survival rate of SPIs in one or two interventions was calculated by counting failure events among the total number of SPIs placed in a single study with a 95% confidence interval (CI); this was represented by a forest plot. A Cochran’s Q test and an I^2^ test were applied to determine statistical heterogeneity. Analyses were performed using Stata version 15 software (Stata Corp., College Station, TX, USA).

## 3. Results

### 3.1. Study Selection

The initial electronic database search found 2571 articles and the manual search identified an additional 4 (n = 2575). Of these, 1286 were duplicates or triplicates and were excluded. After a first scan to discard articles unrelated to the PICO(s) question, followed by title and abstract screening, 29 articles were selected for full text analysis. A total of 15 of these were excluded because they did not meet the inclusion criteria (Table A1 from Appendix A).

**Figure 1:**
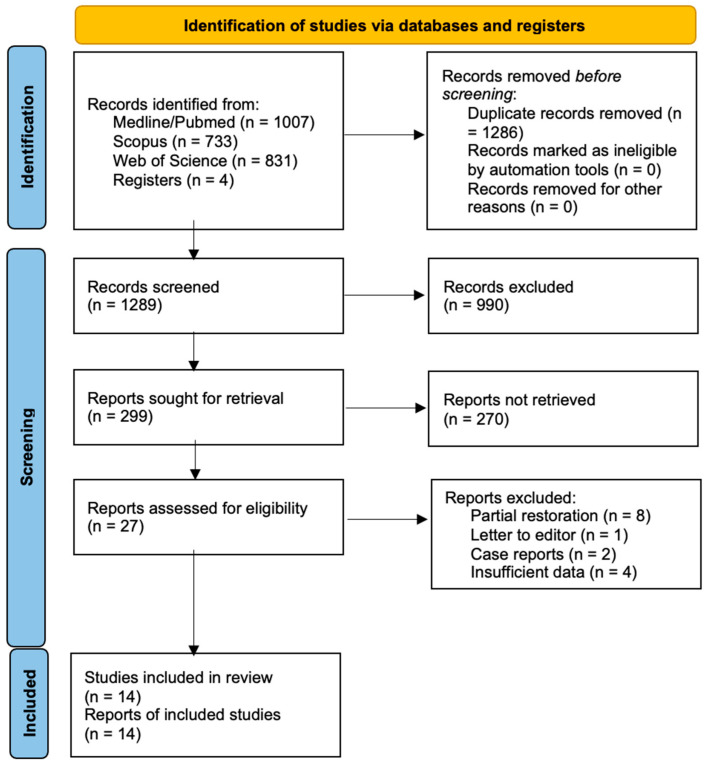
Flowchart illustrating the selection process.

Finally, 14 studies underwent review and data extraction: 4 prospective studies [8,20,21,22]; 5 retrospective studies [10,23,24,25,26]; and 5 case series studies [11,27,28,29,30]. Of the 14 articles reviewed, 5 were performed with a single surgical intervention [8,9,11,20,30] and 9 with two surgical interventions [21,22,23,24,25,26,27,28,29]. The flow diagram in Figure 1 illustrates the search and selection process in detail.

### 3.2. Inter-Investigator Agreement

The Cohen’s Kappa statistic between the two reviewers (L.S.L and S.B.B) was 0.921 (CI 95% 1.032–0.810) for the title and abstract selection and 0.949 (CI 95%: 1.060–0.837) for the full text assessment, pointing to an almost perfect agreement level. Intervention by the third reviewer was not required [31].

### 3.3. Study Characteristics

Table 1 and Table 2 show basic information about the articles—study design, number of patients, number of implants placed, their position and survival, follow-up periods and complications. The minimum number of patients included per study was four and the shortest follow-up was 4 months.

### 3.4. Synthesis of Results

#### 3.4.1. Patient Characteristics

The studies were divided into two groups: one or two surgical interventions for SPI placement. Five studies described single surgical interventions, including a total of 61 SPIs in 46 patients, who were restored with 61 fixed dental prostheses (FDP).

All studies placed one subperiosteal implant to support an FDP, with the exception of Van den Borre et al. [16], who placed two implants per maxilla to restore 15 maxillae.

As for implant loading, SPIs were loaded immediately in two studies [11,20]; early loading was performed in one study surgery (between 48 h and 2 weeks after SPI placement) [10]; loading was performed after healing and soft tissue remodeling in one study [26]. One study did not provide this information [8].

In all the studies, definitive prosthesis placement was carried out between 1.5 months and 4 months after implant placement, with the exception of Elsawy et al. [30]. who placed the definitive prosthesis 12 months after surgery.

The studies performing two surgical interventions placed a total of 912 SPIs in 912 patients, restoring them with 912 FDP. None of these studies stated the precise moment of definitive prosthesis placement.

The total number of SPIs across the studies reviewed was 973 placed in 958 patients, with ages ranging from 39 to 90 years. Five studies did not state the sex of the participants [22,24,25,27,28], while the other nine studies included 216 women and 90 men.

#### 3.4.2. Subperiosteal Implant Survival Rate

When the implant survival rate of subperiosteal implants placed in single surgical interventions was calculated (Cochran’s Q (df = 0); *p* (value) = 1.000; I^2^ = 0%), no statistical heterogeneity was detected. But statistical heterogeneity was detected for the implant survival rate of subperiosteal implants placed in two procedures (Cochran’s Q (df = 8) = 129.34; *p* (value) = <0.001; I^2^ = 99.3%).

**Figure 2:**
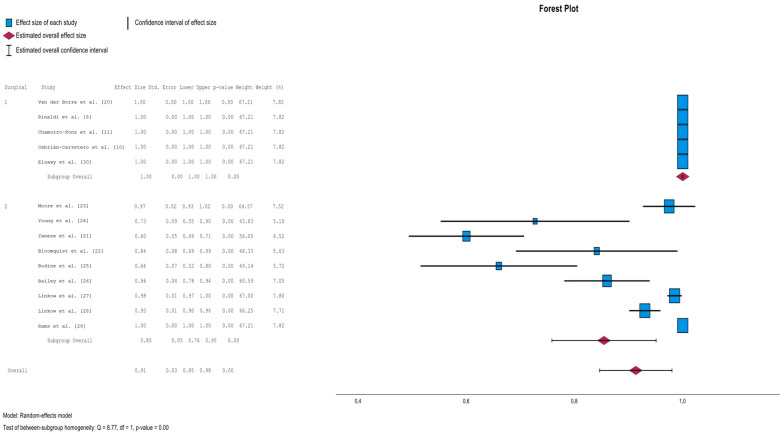
Forest plot for survival rate [8,10,11,20,21,22,23,24,25,26,27,28,29,30].

As heterogeneity between the two groups was observed (Cochran’s Q (df = 1) = 8.77; *p* (value) = <0.001; I^2^ = 100%), it was decided to create a random-effects model. In meta-analysis, it was found that the overall survival rate of subperiosteal implants placed in one surgical intervention was 100%, 95% CI (with follow-up periods of between 4 and 38 months), while the overall survival rate of SPIs placed in two interventions was 85%, 95% CI (76–95%) (with follow-up periods of between 3 and 22 years). The survival rate of the two groups together was 91%, 95% CI (85–98%). The difference between one and two surgical interventions was statistically significant *p* (value) < 0.001 (Figure 2).

#### 3.4.3. Complications

A total of 10 out of the 14 studies reported complications. Articles in which SPIs were placed in one surgical intervention reported three implant exposures and one case of gingival inflammation [8,11,30]. Two studies reported no complications [9,20].

Five studies placing SPIs in two surgical interventions reported the following complications: 34 implant exposures, 45 paresthesias, 13 cases of gingival inflammations, three cases of peri-implantitis, five bone screw sequestrations, three epuli, and three fractures of the definitive prosthesis [21,22,24,25,26,27,29]. One study reported no complications [19] and three studies did not provide information about complications [21,22,28].

The overall complication rate for single-surgery studies was 8.70%, and for two-surgery studies 20.87%.

#### 3.4.4. Quality Assessment of Individual Studies

The Newcastle–Ottawa scale provided quality assessment of cohort studies. All scored four to six stars (Table 3), six stars indicating medium-level quality, and five stars or fewer indicating low quality [32]. The Joanna Briggs Institute Critical Appraisal tool was applied to assess case series, with scores ranging from 5 to 8 points (Supplementary Material Table S2).

## 4. Discussion

This SR set out to evaluate and compare the clinical results—survival rate and complications—of SPIs supporting full arch rehabilitation of edentulous jaws placed in either one or two surgical procedures. A total of 14 clinical studies were included: eight cohort studies and six case series. Five of these studies performed a single surgical intervention to place a total of 61 SPIs, while the remaining nine studies performed two surgical interventions to place 912 SPIs.

The continuous increase in life expectancy coupled with concomitant deterioration of the stomatognathic system causes an increase in the number of geriatric patients with edentulous jaws. Although conventional osseointegrated implants achieve high levels of success [33,34], good survival rates, and boost patient satisfaction [34,35,36], some cases involve large bone defects, associated with extensive peri-implantitis processes that will demand complex reconstructive surgery before implant placement can take place.

Large bone defects also occur in patients suffering from maxillomandibular mutilations, due to tumors or severe cranioencephalic trauma. Clinical scenarios such as these call for alternative graftless treatments. In recent years, it has been argued that zygomatic implants offer an effective option for treating severe maxillary atrophy, obtaining excellent survival rates (98.7% after 46.5 months’ follow-up) [37,38,39,40]. However, this technique suffers several limitations and drawbacks, and zygomatic implants should not be placed in cases presenting a lack of bony support of the malar bone. Zygomatic implant placement can also trigger complications including infection of the implant apex, gingival retraction, communication between the oral cavity and the maxillary sinus, sinusitis (4.7%), extraoral fistulas, and intraorbital abscesses [10]; some of these may prove difficult to manage [41]. And obviously, their use is limited to the maxilla. In cases of zygomatic implant failure caused by a lack of osseointegration, alternative solutions are severely limited due to the resorption of the zygomatic bone following its removal. This is less problematic in cases of SPI failure, as clinicians can place a new SPI after a 3-month healing period [42].

The SPI was designed to distribute prosthetic stress to larger areas of supporting bone, making the SPI a more conservative option for full arch rehabilitation of extremely resorbed jaws [43]. At the same time, by stabilizing SPIs superficially, there will be a reduced risk of injury to adjacent nerve structures [44]. Their placement is minimally invasive, associated with reduced surgical morbidity [45].

SPI use has spread to other clinical/surgical specialties such as oncological or craniofacial trauma patients needing reconstructive/craniomaxillary surgery to restore, for example, orbital or mandibular structures [9].

The primary objective of the present SR was to evaluate the survival of SPIs supporting full-arch restorations of edentulous jaws. The overall survival rate of the two groups (one or two surgical interventions) was 91% (CI 95%: 85–98%), with statistically significant difference between the groups (*p* < 0.001). SPI placement in a single intervention yielded a survival rate of 100%, dropping to 85% (CI 95%: 76–95%) when placed in two stages. This difference in survival rate can be attributed to several factors such as the difficulty of adapting the structures in two-stage procedures, the different material used to manufacture the SPIs and the shorter follow-up times reported in the single-stage cases published to date. Regarding the material used, SPIs placed in two surgical interventions were not made of titanium but, for example, of vitallium [24], this alloy being less biocompatible than titanium, with decreased biomechanical fixation and increased intra- and extracellular accumulation of metal ions in the area immediately surrounding the implant [46].

The secondary objective of this SR was to investigate complications arising from the technique. It should be noted that fewer complications were observed in the studies that performed SPI in a single phase compared with two phases (8.70% vs. 20.87%). Moreover, these complications were more severe in the two-stage SPI procedures. A total of 45 paresthesias were observed in studies with two surgical interventions, but no case of paresthesia was reported for single surgical interventions. Although the present review included many more two-stage surgeries than single interventions (912 vs. 61), this significant difference in complication rates can be explained by the fact that—thanks to advances in digital imaging—a single surgery procedure for the fabrication of the SPI is possible, making use of CAD-CAM technology to design the structures by superimposing CBCT files and intraoral scans of the patient [11]. Nevertheless, it is true that the digital resources (devices and software) required to design and manufacture SPIs are not accessible to all professionals [47].

One of the major biases of this review has been having to join prospective studies with case series in studies using SPIs in a single surgery. This was due to the small number of studies published and to try to reach a unified result. It should be noted that the studies that use two surgical interventions seem to be in disuse, since the studies are prior to the year 2000. These studies are the ones with the largest number of patients and the longest follow-up, so that, despite the good results observed in full arch rehabilitations supported by SPIs, especially those placed in a single surgery, the present SR suffered several limitations. Higher-quality studies are needed, as the studies reviewed here suffer from important methodological flaws. Ideally, studies of single-stage SPIs should have longer follow-up times. Of course, the adaptation of SPIs to new digital imaging techniques is a relatively recent development and so sufficient time has not yet lapsed to allow extensive follow-up times. Lastly, randomized clinical trials of parallel design comparing different techniques (for example, SPIs vs zygomatic implants) could yield more robust data about the clinical outcomes of SPIs. Therefore, in our view, future lines of research should be randomized clinical trials using the support of new technologies and new materials, because it seems that thanks to this, SPIs have a higher success rate and a lower number of complications.

## 5. Conclusions

While noting this SR’s limitations, we may conclude that:

- SPIs would appear a good option for full-arch rehabilitation of severely resorbed edentulous jaws. The studies included in this SR obtained high survival rates and a low rate of complications, especially for those SPIs placed in a single surgery.
- It would appear that applying CAD-CAM technology to the design of these structures, and so reducing the procedure to a single surgery, improves outcomes and minimizes complications.
- Nevertheless, we should interpret the results of this SR with some caution; well-conceived clinical trials—ideally randomized clinical trials with adequate sample sizes and longer follow-up periods—are needed to confirm our findings.

## Figures and Tables

**Table 1:** Studies evaluating subperiosteal implants in which only one surgical procedure is performed.

Author Year Journal	Study	Patient	Follow-Up	Implant Number Position	Nº of Interventions	Time and Type of Prosthesis Loading	Survival Rate	Complications
Van der Borre et al. [20] 2022 Int. J. Oral Maxillofac	Prospective	15 (8 ♂ 7 ♀)	12 months	30 implants (2 per maxilla) 15 maxillae	1	Immediate loading Definitive prothesis/2 months	100%	No complications
Rinaldi et al. [8] 2020 Ann. Maxillofac. Surg.	Prospective	15 (7 ♂ 8 ♀) (mean age 61 years)	8–19 months	15 maxillae	1	-	100%	2 implant exposures
Chamorro-Pons et al. [11] 2021 Rev. Esp. Cir. Oral Maxilofac	Case series	8 (2 ♂ 6 ♀) (59–82 years)	4–36 months	8 maxillae	1	Immediate loading Definitive prothesis/1.5–2 months	100%	1 gingival inflammation
Cebrian-Carretero et al. [10] 2022 J. Clin. Med.	Case series Retrospective	4 (3 ♂ 1 ♀) (66.2 years)	9–38 months (medium 18 months)	4 maxillae	1	Early loading/14 days Definitive prothesis/1.5 months	100%	No complications
Elsawy et al. [30] 2022 J. Prosthet. Dent.	Case series	4 (2 ♂ 2 ♀) (65–75 years)	12 months	4 maxillae	1	Early loading/After healing and remodeling of the soft tissue Definitive prothesis/12 months	100%	1 implant exposure

**Table 2:** Studies evaluating subperiosteal implant placement performed in two surgical interventions.

Author Year Journal	Study	Patient	Follow-Up	Implant Number Position	Number of Interventions	Time and Type of Prosthesis Loading	Survival Rate	Complications
Moore et al. [23] 2004 J. Prosthet. Dent.	Retrospective	40 (7 ♂ 33 ♀)	18 years	40 mandibles	2	-	97.5%	No complications
Young et al. [24] 1983 J. Prosthet. Dent.	Retrospective	25 (-)	20 years	25 mandibles	2	-	72.7%	3 new prostheses
Yanase et al. [21] 1994 J. Prosthet. Dent.	Prospective	81 (18 ♂ 63 ♀) (39–77 years) 53 years	21 years	81 mandibles	2	-	60%	-
Bloomquist et al. [22] 1982 J. Oral Maxillofac Surg.	Prospective	23 (-)	4.5 years	23 mandibles	2	-	84.2%	-
Bodine et al. [25] 1996 J. Prosthet. Dent.	Retrospective	41 (19 ♂ 22 ♀)	20 years	41 mandibles	2	-	66%	10 exposures 3 epuli 5 sequestration of bone screw 13 gingival inflammation
Bailey et al. [26] 1988 J. Prosthet. Dent.	Retrospective	74 (17 ♂ 57 ♀) (53 years)	14 years	74 mandibles	2	-	86%	38 paresthesias 24 exposures
Linkow et al. [27] 1998 J. Oral Implantol.	Case series	317 (-)	3 years	317 mandibles	2	-	98.7%	7 paresthesias
Linkow et al. [28] 1998 J. Oral Implantol.	Case series	300 (-)	12 years	300 maxillae	2	-	93%	-
Rams et al. [29] 2013 J. Oral Implantol.	Case series	11 (2 ♂ 9 ♀) (64–83 years)	10–13 years (3 subjects) 9–22 years (8 subjects)	11 mandibles	2	-	100%	3 patients with periimplantitis

**Table 3:** Quality assessment of included studies using the Newcastle–Ottawa scale. ★ = 1.

	Selection				Comparability		Outcome			Number of Stars (Out of 8)
Study	S1	S2	S3	S4	C1	C2	E1	E2	E3	
Van der Borre et al. [20]	★	0	★	★	★	0	0	0	★	5
Rinaldi et al. [8]	★	0	★	★	★	0	0	0	★	5
Moore et al. [23]	★	0	★	★	★	0	0	★	★	6
Young et al. [24]	★	0	0	★	★	0	0	★	0	4
Yanase et al. [21]	★	0	★	★	★	0	0	★	★	6
Bloomquist et al. [22]	0	0	0	★	★	0	0	★	★	4
Bodine et al. [25]	★	0	★	★	★	0	0	★	★	6
Bailey et al. [26]	0	0	★	★	★	0	0	★	★	5

## Supplementary Materials

The following supporting information can be downloaded at: https://www.mdpi.com/article/10.3390/dj13060240/s1, Table S1: Search strategy, based on PICO question and MeSh index terms, Boolean terms and its truncations; Table S2: Quality assessment of included studies using the Joanna Briggs Institute Critical Appraisal tools.

## Abbreviations

The following abbreviations are used in this manuscript:

GBR	Guided bone regeneration
SPIs	Subperiosteal implants
SR	Systematic review

## Appendix A

**Table A1:** Excluded articles.

Studies	Reason for Exclusion
Paris et al., 1978; Linkow et al., 1999 Aras et al., 2005; Claffey et al., 2015	Insufficient data
Sirvu et al., 2003; Nguyen et al., 2018	Case report
Knott et al., 2010	Letter to editor
Kay et al., 1987; Minichetti et al., 2003 Gellrich et al., 2017; Cerea et al., 2018; Mittal et al., 2019; Mangano et al., 2020; Onică N et al., 2024; Zielinski R et al., 2025	Partial restoration
