# Supplementary material for: Clinical Performance of Subperiosteal Implants in the Full-Arch Rehabilitation of Severely Resorbed Edentulous Jaws: A Systematic Review and Metanalysis
Source: Dent J (Basel). 2025 May 28;13(6):240. doi: 10.3390/dj13060240 (PMC12191889; doi:10.3390/dj13060240)
Supplement: Supplementary file 1 [file dentistry-13-00240-s001.zip › dentistry-3601654-supplementary.pdf]

Supplementary material Table S1. Search strategy, based on PICO question and MeSh index terms, Boolean terms and its truncations.

Focused question (PICO)	In edentulous patients with severe atrophy of the jaws restored with subperiosteal implants, what is the clinical performance (in terms of survival rate and complications) of SPIs differentiating between single-phase and two-stage procedures?	
P (Population)	Edentulous patients with severe atrophy of the jaws restored with subperiosteal implants.	1# (Edentulous OR partially edentulous)
I (Intervention)	subperiosteal implants supporting full-arch rehabilitations placed in a single surgery	2# (subperiosteal implants) AND (single surgery OR one surgery)
C (Comparison)	subperiosteal implants placed in two surgical procedures.	3# (subperiosteal implants) AND (double surgery OR two surgery)
O (Outcome)	Clinical performance in terms of survival and complications.	4# (Survival rate OR complication OR outcome)
Search combination PubMed, Web of Science and Scopus	1# AND 2# AND 3# AND 4#	
Terms truncation PubMed, Web of Science and Scopus	(Edentulous OR partially edentulous) AND (subperiosteal implants) AND (single surgery OR one surgery) AND (subperiosteal implants) AND (double surgery OR two surgery) AND (survival rate OR complication OR outcome)	

Supplementary material Table S2. Quality assessment of included studies using the Joanna Briggs Institute Appraisal tool.

**Study**	Chamorro-Pons et al.[11]	Cebrián-Carretero et al.[10]	Linkow et al.[23]	Linkow et al.[24]	Rams et al.[25]	Elsawy et al.[26]
**Were there clear criteria for inclusion in the case series?**	+	?	-	+	?	+
**Was the condition measured in a standard, reliable way for all participants included in the case series?**	+	+	+	+	+	+
**Were valid methods used for identification of the condition for all participants included in the case series?**	+	+	+	+	+	+
**Did the case series have consecutive inclusion of participants?**	+	+	?	-	-	?
**Did the case series have complete inclusion of the participants?**	+	+	?	?	?	+
**Was there clear reporting of the demographics of the participants in the study?**	+	+	+	+	+	+
**Was there clear reporting of clinical information of the participants?**	+	+	-	+	+	+
**Were the outcomes or follow up results of cases clearly reported?**	+	+	+	+	+	+
**Was there clear reporting of the presenting site (s)/clinic (s) demographic information?**	-	+	+	+	-	+
**Was statistical analysis appropriate?**	-	-	-	-	+	-
**Overall appraisal**	Included	Included	Included	Included	Included	included

+=Yes; - =No; ?=Unclear
